# One lung ventilation management in a patient with Reinke’s edema

**DOI:** 10.1016/j.ijscr.2020.04.097

**Published:** 2020-05-17

**Authors:** Marco Rispoli, Moana Rossella Nespoli, Dario Maria Mattiacci, Carlo Curcio, Dino Casazza, Dario Amore

**Affiliations:** Monaldi Hospital, Naples, Italy

**Keywords:** Reinke’s edema, VivaSight-endoblocher™, VivaSight SLT

## Abstract

**Introduction:**

Reinke’s edema (RE) is a benign laryngeal disease. We describe the case of a patient with history of bilateral RE requiring surgical treatment, that came to our attention for a lung lobectomy due to adenocarcinoma. In consideration of the possible complications at the time of extubation and of the probable difficult control of the airways, the patient underwent intervention of microflap surgery for the RE at the same time of lobectomy. We opted for Bronchial Blocker (BB) using a Viva-sight™ Single Lumen Tube (SLT) Internal Diameter (ID) 7.0 mm (Ambu A/S, Baltorpbakken 13, DK-2750 Ballerup, Denmark) with integrated high-resolution camera.

**Presentation of case:**

The patient (female, 67 years old, BMI 28) was a candidate for lung lobectomy. She reported a RE requiring surgical treatment. An armoured Endo Tracheal Tube (ETT) ID 5.0 mm was positioned and microflap surgery was performed. Once this surgery ended, the armoured ETT was removed after placing an airway guide wire exchanger and a SLT ID 7.0 mm was placed. ​VivaSight-endoblocher™ (EB) was positioned in the right bronchus.

**Discussion:**

We opted for double intervention, the risk that could result from the delay persuaded the patient to perform surgery for the RE. Postponing the lobectomy was dangerous for the oncological situation. The Viva-Sight SLT represented the right compromise.

**Conclusion:**

Even after the microflap, the space available for the ETT was reduced and, in order not to traumatize a tissue already stressed by surgery and to facilitate the tracheal intubation, we opted for BB using a Viva-sight™.

## Highlights

• Reinke’s edema.
• VivaSight Single Lumen Tube.
• VivaSight BB.
• VATS lobectomy.
• Thoracic surgery.

## 1 Introduction

Reinke’s edema (RE) is a benign laryngeal disease that is often associated with chronic irritation from smoking, leading to edema and polypoid degeneration of the true vocal cords. The tipical clinical manifestation is characterized by nonspecific clinical features such as dysphonia and hoarse-ness of voice. Despite the benign nature of this edema, anesthesiologists should be aware of this condition as it may complicate airway management during tracheal intubation and extubation. Indeed RE often narrows the glottic aperture, hindering endo-tracheal tube (ETT) passage. Small size ETT may be needed to achieve succesful intubation [1]. Furthermore, the reactive edema due to airway instrumentation and prolonged tracheal intubation is added to RE to produce clinically life-threatening airway obstruction after tracheal extubation [2].

The potentially life-threatening condition of RE patients is of particular concern in case of lung surgery requiring One Lung Ventilation (OLV) due to the large diameter of the Double-Lumen Tubes (DLTs) commonly used in such surgery that could more commonly cause reactive edema. Actually the optimal size of the DLTs is the largest tube whose bronchial lumen fits the main bronchus with only a small air leak when its bronchial cuff is not inflated, so the tendency to use larger diameters is common [3]. Even in case of Bronchial Blocker (BB) use, the room needed for bronchoscope and for BB itself often force the anesthesiologist to use a larger ETT compared to the patient anthropometry [4].

RE reatment is focused on decrease of risk factors, such as smoking cessation, voice therapy, and reflux control while surgical techniques aim to decrease redundant polypoid mucosa in order to improve voice and restore the glottic airway [5].

We describe the case of a patient with history of bilateral RE requiring surgical treatment, that came to our attention for a lung lobectomy due to adenocarcinoma. In the past years she refused any treatment options and surgery for her RE, until severe dyspnea develops. In consideration of the possible complications at the time of extubation and of the probable difficult control of the airways, the patient underwent intervention of microflap surgery for the RE at the same time of lobectomy. Even after the microflap was performed, the space available for the ETT was still seriously reduced so, in order not to traumatize further a already stressed by surgery tissue and to facilitate the tracheal intubation, we opted for BB using a Viva-sight™ Single Lumen Tube (SLT) ID 7.0 mm (Ambu A/S, Baltorpbakken 13, DK-2750 Ballerup, Denmark) with integrated high-resolution camera.

The work has been reported in line with the SCARE criteria [6].

## 2 Presentation of the case

**Fig. 1:**
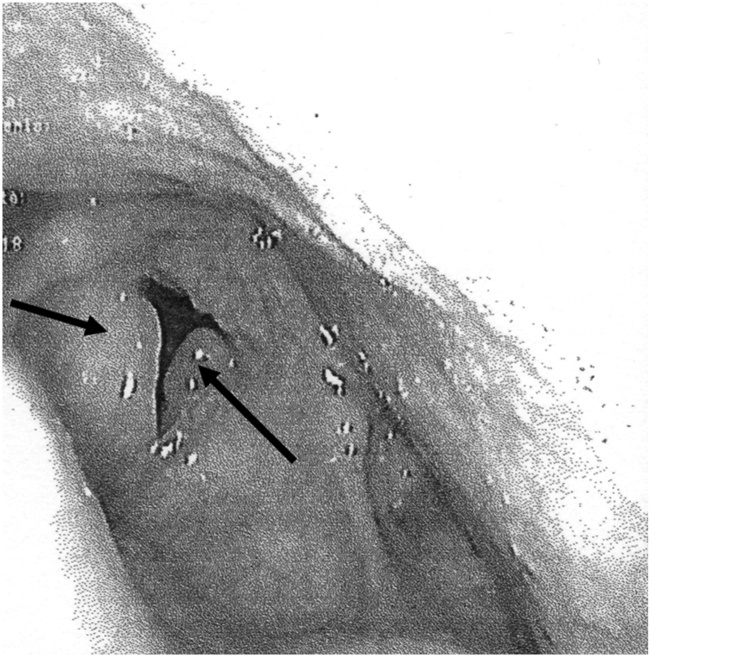
Endoscopic image showing edematous vocal cords (arrows).

The patient (female, 67 years old, BMI 28) come to our attention for lung lobectomy. In medical history she reported 75-pack-year smoking, a RE requiring surgical treatment, hypertension and coronary artery disease treated with PTCA and stenting. As pre-operative assessment she performed a spirometry: FVC 138% and FEV1 102%; a Diffusing Lung Capacity Carbon Monoxid test (DLCO): 56% and a Blood Gas Analysis (BGA): pH 7.4, pCO2 55 mmHg, pO2 68 mmHg. Preoperative assessment required fibro laryngoscopy showed bilateral lesions with expanded polypoid lesion and confirmed a bilateral grade III RE requiring microflap surgery (Fig. 1). Possible difficult airway was assessed: mouth opening 3.5 cm, thyromental distance 7 cm, Mallampati grade III, good neck mobility, no history of previous tracheal intubation. Blood tests were compatible with age and pathological status.

A multidisciplinary session with anaesthesiologist, otolaryngologist and thoracic surgeon was organized to inform the patient of her health conditions, oncological prognosis and any complications related to RE. Only at the end of this multidisciplinary meeting, patient was asked for informed consent.

**Fig. 2:**
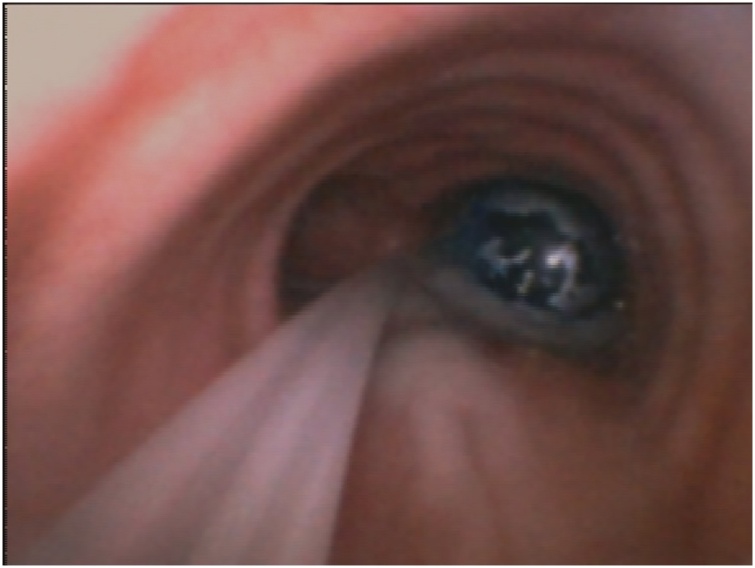
Tracheal carina view through the Viva-sight™ Single Lumen Tube integrated camera, the VivaSight-endoblocher™ is positioned in the right bronchus.

The day of surgery patient was pre-medicated with benzodiazepine due to strong night anxiety. Continuous ECG and Pulse oximetry were set and, after local anesthesia, a left radial artery catheterization was performed to monitor arterial blood pressure. Once preoxygenation was achieved, propofol and remifentanil target control infusion (TCI) anesthesia induction started, using bispectral index (BIS) to assess anesthesia level. Manual ventilation was performed so rocuronium was administered to achieve neuromuscolar blockade. After deep curarization (Train of Four – TOF < 2) a videolaryngoscopy was performed revealing a Cormack-Lehane grade 2A. An armoured ETT ID 5.0 mm was positioned and suspension laryngoscopy for microflap surgery was performed. Once surgery ended, the armoured ETT was removed after placing an airway guide wire exchanger and a SLT ID 7.0 mm was placed. ​VivaSight-endoblocher™ (EB) (Ambu A/S, Baltorpbakken 13, DK-2750 Ballerup, Denmark) was positioned in the right bronchus (Fig. 2). Video Assisted Thoracoscopic Surgery right lobectomy was performed without any complications. Tracheal extubation was carried out after placing an airway guide wire exchanger. No airway complications occurred and patients did not report stridor or dyspnoea once in spontaneous breathing. Four day after surgery patients was dismissed on strict voice rest for 2 weeks, acid reflux medication was also prescribed to prevent stomach acid from damaging the vocal folds as they heal.

## 3 Discussion

Because of the common etiological factor of smoking, patients with laryngeal pathology run an especially high risk of developing lung cancer [7]. The RE is even particularly insidious as it is a pathology often unknown or underestimated by many anesthesiologists but should be considered among causes of postextubation stridor without other causes, especially in patients with risk factors (smoking habit, gastro-oesophageal reflux and voice abuse) [2].

Our patient, despite being aware that her RE required surgical therapy, has always avoided surgery due to fear, also in consideration of her still good respiratory performances. When she came to our attention she was focused on the lung surgery, not expecting the RE to expose her to a serious risk for the airway management. Therefore the patient would have firmly consented to the microflap surgery for RE only to undergo the lobectomy. This was the reason why we opted for double intervention, the risk that could result from the delay persuaded the patient the patient to perform surgery for the RE before. Postponing the lobectomy was dangerous for the oncological situation of the patient.

The main concern in this case was related to a possible deterioration of the RE due to the prolonged decubitus of a large ETT, such as DLT necessary to perform OLV. The patient’s anthropometry would have required the positioning of a 37 Fr DLT (14 mm Outer diameter) which would surely cause decubitus on the vocal cords, furthermore DLTs’ elliptical cross-section with a larger external diameter on the lateral aspects (compared with single-lumen tubes), increases the potential risk for traumatic injuries to the airway [8].

On one hand the use of a BB with small-calibre ETT, such as the ID 7 mm, would have guaranteed less traumatism to the airways thanks to the much smaller outer diameter (9.6 mm) but on the other hand, possible complications could depend on the internal diameter. In fact, most bronchial blockers have a diameter of about 3 mm which occupies almost half the internal diameter of a ETT ID 7 mm, making it difficult to perform a bronchoscopic guidance in case of difficult positioning.

Therefore the Viva-Sight SLT represented the right compromise due to its integrated high-resolution camera granting continuous monitoring of ETT and EB position. The small size of the ETT combined with the advantage of not having to use a bronchoscope arrowed to adopt the least traumatic solution for the airways.

Despite all precautions in airway management, microflap surgery has been considered necessary in any cases because even a small ETT could be the trigger for postextubation worsening edema considering the high RE grade. One aspect beyond the clinical management is that this case has given clinicians awareness of this pathological condition which is often underestimated despite the risk factors common to lung cancer.

## 4 Conclusion

Even after the microflap, the space available for the ETT was reduced and, in order not to traumatize a tissue already stressed by surgery and to facilitate the tracheal intubation, we opted for BB using a Viva-sight™ SLT. Not having to use a bronchoscope for BB guidance granted us to choose the smaller ETT possible, without excessively traumatizing the vocal cords.

## Declaration of Competing Interest

No author has conflict of interest.

## Funding

This research did not receive any specific grant from funding agencies in the public, commercial, or no profit sectors.

## Ethical approval

The ethical approval has been exempted by my institution.

## Consent

Written informed consent was obtained from the patient for publication of this case report and accompanying images. A copy of the written consent is available for review by the Editor-in-Chief of this journal on request.

## Author contribution

Conception and design of study: M Rispoli, C Curcio.

Acquisition of data: MR Nespoli, D Casazza, D. Amore.

Drafting the manuscript: D M Mattiacci.

Revising the manuscript critically for important intellectual content: MR Nespoli, M Rispoli.

Approval of the version of the manuscript to be published M Rispoli.

## Registration of research studies

NA.

## Guarantor

Rispoli Marco.

Nespoli Moana Rossella.

## Provenance and peer review

Editorially reviewed, not externally peer-reviewed.
